# Supplementary material for: Chromosomal DNA sequences of the Pacific saury genome: versatile resources for fishery science and comparative biology
Source: DNA Res. 2024 Mar 7;31(2):dsae004. doi: 10.1093/dnares/dsae004 (PMC11090075; doi:10.1093/dnares/dsae004)
Supplement: dsae004_suppl_Supplementary_Tables_S1-S4_Figures_S1-S4 [file dsae004_suppl_supplementary_tables_s1-s4_figures_s1-s4.docx]

**Supplementary Information**


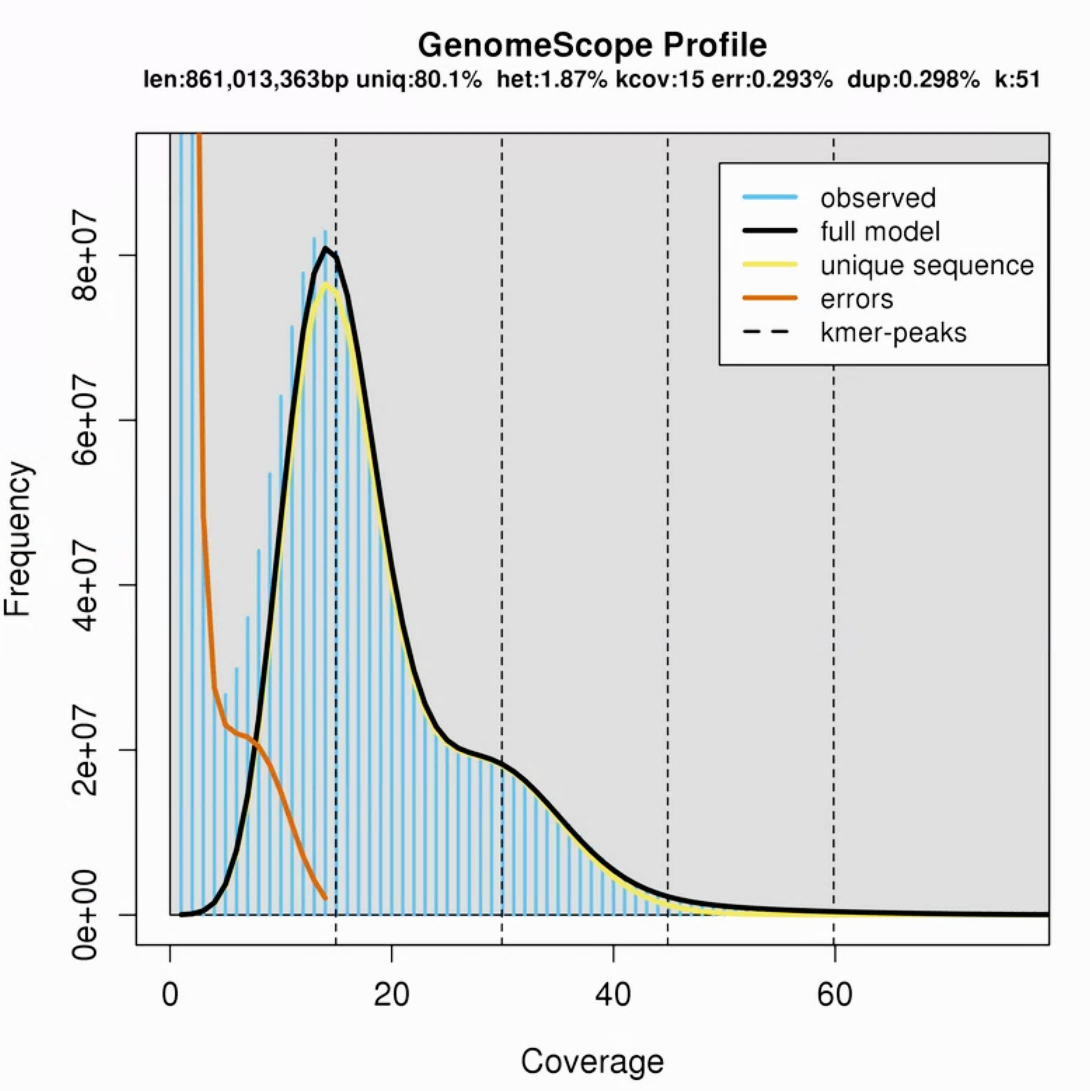


**Supplementary Figure S1**. Distribution of k-mers based on HiFi reads. K-mer frequency was computed with the jellyfish v2.3.0, and the resultant .histo file is input in the GenomeScope^1^.


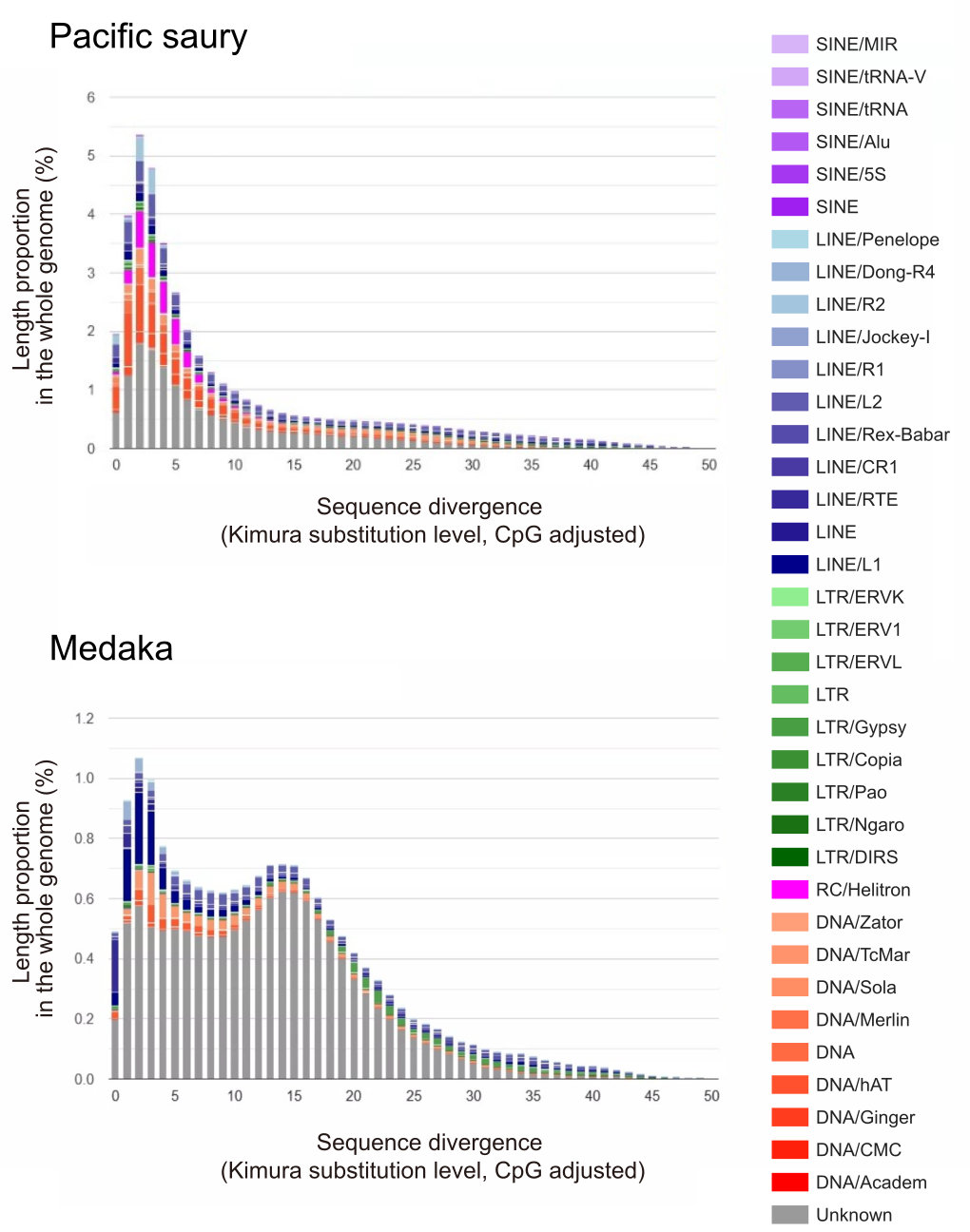


**Supplementary Figure S2**. Repetitive element compositions and their divergence profiles. Distribution of sequence divergence of the identified repetitive elements. The divergence of repetitive elements in individual repeat subclasses was computed using the Perl scripts, calcDivergenceFromAlign.pl and createRepeatLandscape.pl, that are accompanying the program RepeatMasker. See Materials and Methods for details of repeat detection. Note that the height is not equally scaled between the species.


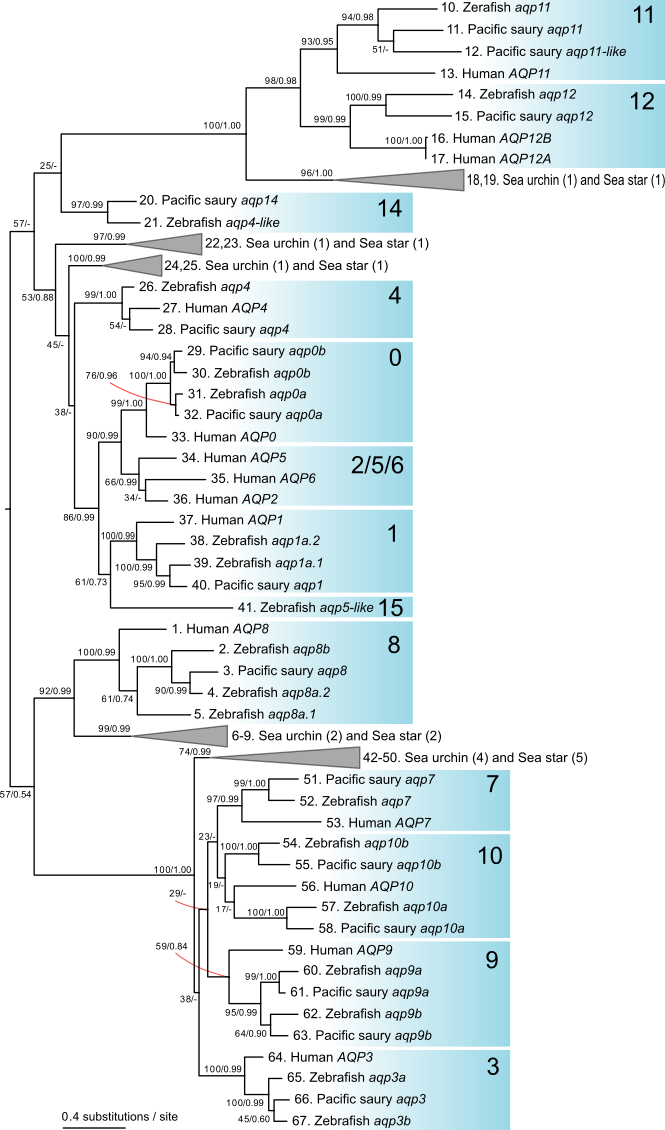


**Supplementary Figure S3**. Aquaporin gene phylogeny. Phylogenetic tree confirming the orthology of the Pacific saury genes. The maximum-likelihood tree was inferred with 232 residues in the amino acid sequence alignment (see Materials and Methods). The support values at notes are bootstrap probabilities in the ML tree and posterior probabilities in the Bayesian inference (see Materials and Methods). The numbers in the brackets shown the multiplicity of the sequences used for the species indicated.

**
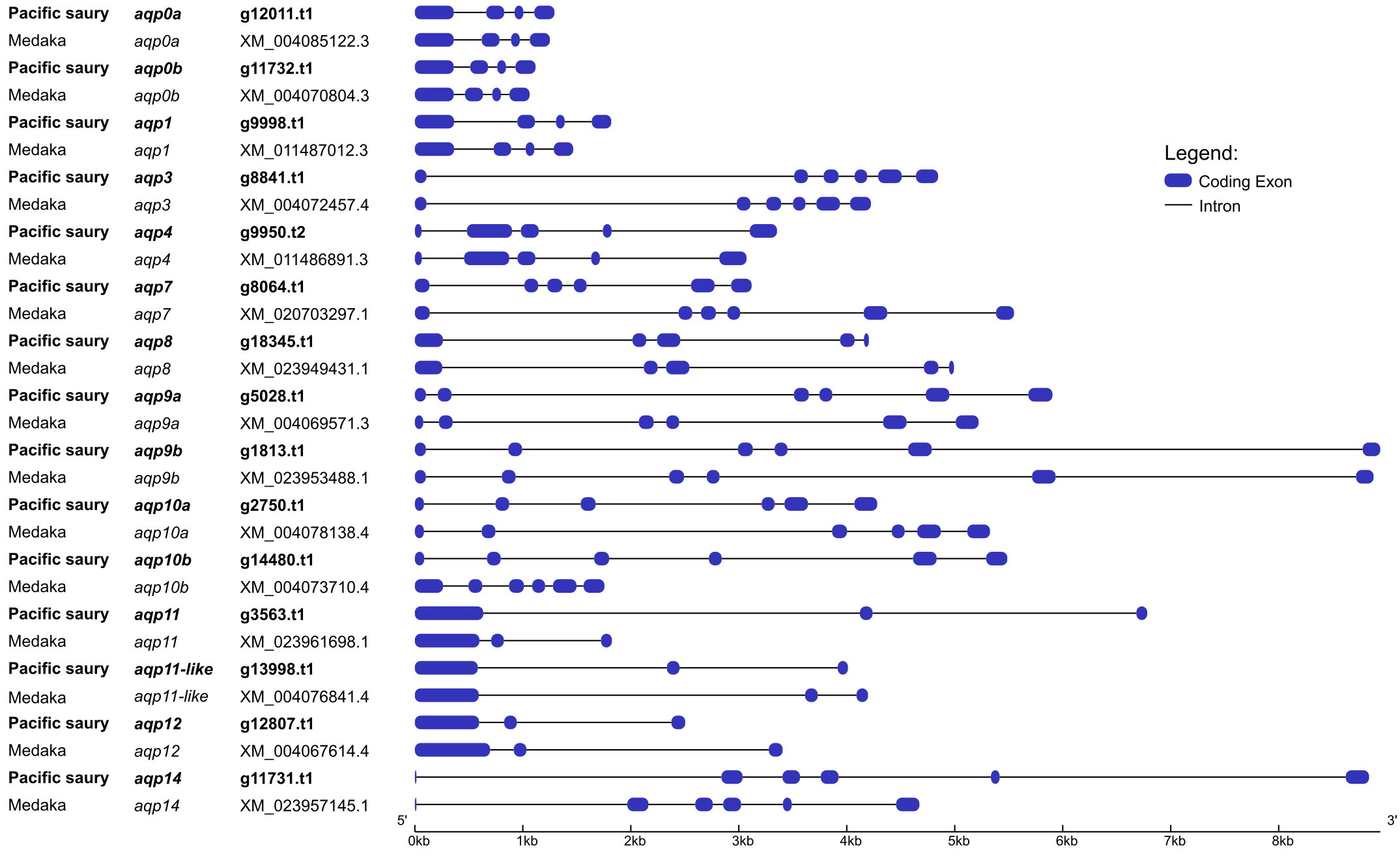
**

**Supplementary Figure S4.** Exon-intron structure of Pacific saury Aquaporin (aqp) genes. The protein-coding exon organization was compared with that of medaka, using GSDS 2.0 software^2^. Boxes represent protein-coding exons, and lines denote introns. The primary structure of each gene is similar between the species, with an exception of *aqp10b*. The Pacific saury *aqp10b* gene has much longer introns compared with the medaka ortholog, and the first coding exon of the Pacific saury *aqp10b* gene is much shorter than that of its medaka ortholog, due to a putative misidentification of the start codon which allegedly causes an extended N-terminus of the gene product in medaka. Pacific Saury transcript IDs and medaka GenBank IDs are provided alongside the gene names. Note that the teleost fish *aqp11* gene nomenclature remains to be phylogenetically resolved to adapt to the typical a/b format.

| **Supplementary Table 1.** Nucleotide and deduced amino acid sequences of the transcripts used for the analysis. | | |
| --- | --- | --- |
| Gene | Type | Sequence |
| ACAT1 | Nucleotide sequence | >TRINITY_DN3704_c0_g1_i1 AAGGTTTGAGCCCCAGCCCCCCCCAGTCATCTGTCGTTGTGTCCTTGGGCGAGACACCTCACCGTCCTTGCCTCCAGTGAGGGCATCGCTGGTGTGTGAATGTGTATGAACGTTCCGGTCGGAGAGCTGGTTAGGCGGAGATCGGCTGCCAGTCTTCCGTCAGTCTGCCCCAGGGCAGCTGTGGCTACACATGTAGTTTACCATCATGAGGTGTGAATGGATAATGGAACCATTGTGAGCACTTTGAGTGTCCAGAAAAGCACGATATAAATGTAATCCATTAGTAAAAATATGTTCAAACATGTGTACCATAAAAGGCATCTCTGGCATGCTTTGGTCTCTCCTGCAATAGCAGCAACGGAAATTAAAACAAGCTAAATGTCGCCATCTAGTGGACAATAATCGAACTGTATCACCCACACCAAAATAAGTTTCACCTTTAACCCACACCGACAGGTGACAAGACAGTTTTCATGAAAAACTATTTATTCAACCACATCAAGTTAAATATTAATATTTTTTTCTATCAAAGAAAGGAGTGTATGGTACACAGCATTCAAGTCAGAAAAGTTGAGGAGTTGCCTGAAAACAGGCTGGAGAGTTAAGGACCGCTGGCAGAAGTGGCAACATTCATTCGCTATAGTTATGAGTGTACAGAAGTCTTAACTATTTAGAACAGAGTCTGTCACAGCAAAATGTAACAAAGAGTGCATCAGAGAACAAGTGTGCTTCTCTCTGCTTTGCTTAAAACTTCTGGATCAGGATAGAGGAGGCTCCACCGCCTCCGTTGCAGATGCCTGCCAGCCCGTACTGACCTGCCTTCAGGTTGTGAACCATGTGACCCACAATTCTCGCCCCAGACATCCCAATAGGGTGTCCCAGAGACACAGCCCCCCCGTTGACGTTGACCTTCGCAGGGTCAATATGGAGCATCTTGATGTTGGCCAGCACGACCACGCTGAAGGCCTCGTTGATCTCCCACATATCAATATCTTCCTTCTTCAAGCCCGCTGCACTCAGCACCTTTGGCACAGCATATGCAGGAGCGATGGGGAAATCAATGGGTGCAACCGCAGCATCAGCAAAAGAGACAACCCTGGCCAGTGGAGTGACGTTGAGTCTCTTCGCAGCATCTGCTGTCATTAAAACGACAGCTGCAGCTCCGTCGTTCAGAGTGCTGGCGTTGGCCGCCGTCACCGTGCCGTTCTCTTTCTGGAAGACGGCCTTCAGTTTCGGGACTTTGCTGAAGTCGACCCGCCTCCACTCCTCATCCTCAGACACCACAACATCAGGTTTGCCTCTCTGGGGAATGGAAACGGGTACGATCTCCTTGGCCAGCAGGCCGGCCTCGAACGCTGCTTTGCTGCGGGTGTATGAGCTGATGGCGTAGGCATCCTGCTCTTCTCTGCTGATACTGCAGGTCTTTGCAGTGTTCTCTGCACAGTTGCCCATGTGGAATTTGTTGTAGACATCAGTGAGTCCATCCTTGACGATGAGGTCTTCCATCTTCACTCCTCCATAGGATGGGGTGTCTCTGGTCATCACATAAGGAACGTTGGACATGCTCTCCATGCCTCCTGCCACCATTACATCCTGATGTCCACACATTAGACTCTGAGCTGCCAGCATGATGGACTTCATCCCAGAAGCACACACTTTGTTGACGGTTGTTGCTGGAGTGCTGAGGGTCAAACCTGCTCCAAGCAAGGCCTGCCGCGTGGGAGCCTGTCCTTCTCCTGCCTGAAGCACGTTGCCCATGTAAACTTCCTTCACCTCCTCAGGACTAATTCCTGCTCTCTCGATGGCTCCTTTGATGGCGATGGAGCCCAGTTTGGTTGCTGGAACGGCTGCCAGGCTGCTTCTGAAGGAGCCCATTGGCGTGCGGACTGCACTGACAATGACGACTTCATTAAGTGAAGGGCGAGATGTGTAGGTTCTTGAAAGGCACTTGTGAGCCAGGCGTTTACAGATTTGAGCGTGCATGGTTAAACGTCCACAGGATGACATTTTGGACTGGACGACGGGGCTCACGGCCACACTAGCTTCTCCTTCAAGCAGCTGGTGAA |
|  | Deduced amino acid sequence | MSSCGRLTMHAQICKRLAHKCLSRTYTSRPSLNEVVIVSAVRTPMGSFRSSLAAVPATKLGSIAIKGAIERAGISPEEVKEVYMGNVLQAGEGQAPTRQALLGAGLTLSTPATTVNKVCASGMKSIMLAAQSLMCGHQDVMVAGGMESMSNVPYVMTRDTPSYGGVKMEDLIVKDGLTDVYNKFHMGNCAENTAKTCSISREEQDAYAISSYTRSKAAFEAGLLAKEIVPVSIPQRGKPDVVVSEDEEWRRVDFSKVPKLKAVFQKENGTVTAANASTLNDGAAAVVLMTADAAKRLNVTPLARVVSFADAAVAPIDFPIAPAYAVPKVLSAAGLKKEDIDMWEINEAFSVVVLANIKMLHIDPAKVNVNGGAVSLGHPIGMSGARIVGHMVHNLKAGQYGLAGICNGGGGASSILIQKF |
| DLD | Nucleotide sequence | >TRINITY_DN743_c0_g1_i4 GTCACAGAAGCGACGTCCGAGCCATGCGTCCGAGCCCGAGCCGCTGAAGTGGTACTGCGCGTGCGCAATGCCGGTAGTCCTTGGTTGGTTGGCCTGCCGGAGGTGACTCGCCCTGACAGCAACACAGACAACATGCAGAGCTGGACGCCGCTTTACCGCTCTCTGGCCACGCGGGGCCATCATGTCCCCTCCAAACTGCATGGAGCCACAGCTCTGTCTGTCCGGACCTACGCAGACAAAGCTGCAATCGATGCAGATGTCACAGTCGTTGGCTCCGGTCCAGGCGGCTACGTCGCTGCCATAAAAGCTGCACAGCTCGGTTTCAAGACGGTATGTGTTGAGAAAAATGCCACGCTGGGTGGGACCTGTCTCAACGTTGGCTGTATCCCTTCAAAGGCTCTGCTCAACAACTCCTACTTTTACCACCTGGCTCATGGCAAGGACTTTGAAAGCAGAGGCATTGAAATCTCAGGCATCTCGTTGAACCTGGAGAAGATGATGTCTCAGAAGAGCGGAGCCGTCAAGGCGCTGACCGGAGGAATCGCACATCTGTTCAAACAGAACAAAGTAACCCATGTCAGCGGCTTAGGGAGAGTATCTGGTAAGAACCAGGTGACGGCTACAGGCGCTGACGGCAGCGAGCAGGTCATCAACACTAAAAACATCCTCATCGCCACGGGTTCTGAGGTCACACCCTTCCCCGGTATCCACATCGACGAGGAGACCATTGTGTCATCAACAGGGGCTCTGTCCCTGAAGAAAGTGCCAGAAGAGCTGATTGTGATTGGAGCTGGAGTCATTGGAGTGGAGCTGGGGTCAGTGTGGCAACGTCTGGGCTCCAAGGTTACCGCCGTGGAGTTCCTGGGCCACGTTGGAGGAATGGGCATCGACATGGAGATCTCTAAGAACTTCCAGCGCATCCTGCAAAAGCAGGGTCTCAAATTCAAGCTCGGCACCAAAGTCATGGGAGCCACCAAGAGGCCCGATGGCAAGATCGATGTGCCAGTGGAGGCGGCAGCTGGAGGGAAGAACGAGACTCTGACGTGTGACGTGCTGCTGGTGTGCATTGGCAGACGACCCTACACCCAGAACCTGGGTCTGGAAAGTGTCGGCATCGAGTTGGACAAAAGGGGTCGTATCCCTGTCAACAACCGCTTCCAGACCCAAGTACCAAGCATTTATGCGATTGGTGACGTGGTCGCCGGCCCCATGTTGGCCCACAAGGCAGAAGATGAAGGCATCATTTGTGTGGAGGGCATGGCTGGTGGTGCTGTGCACATAGACTACAACTGTGTTCCCTCCGTCATCTACACCCACCCTGAGGTGGCCTGGGTGGGGAAAACAGAGGAGCAGCTCAAAGAAGAGGGCGTCCCATATAAAGTGGGAAAGTTTCCCTTCGCAGCCAACAGTCGAGCCAAGACCAACGCCGACACTGACGGCTTAGTGAAAATCCTCAGTCACAAGGAGACGGACAGGATGCTCGGATCTCACATCCTCGGCTCTGGGGCAGGTGAGATGATCAATGAAGCTGCACTGGCCATGGAGTACGGCGCCTCCTGTGAGGACGTTGCCCGGGTCTGCCATGCCCATCCGACGGTGTCGGAGGCGTTCAGAGAAGCCAACCTGGCCGCTTCCTTTGGCAAAGCCATCAACTTTTGATCTAATAGCCGCTGTCGCACCTTCATGGGTCCAGGAACGGGAGGCTGCGTTTCTGCATCAGCCCGCCGGGATCCTCTGTACATCCAACCTGCACCACACTATGCTTAATGTTATTTAAATGTTATCAATAAAAAAGATGAAACAGCTTAGGTGGTAAATTAATGGTGACTGAAGTCAGTCTTTTTTGTTTTCCTCACATAGTAAAAAAACATCCCAGAGGATTATATTCTGCACTTCCTTTACGGTGCAAAGAGAGAGAGATTTTGACGTGTGGTTTCTTTGACAGGAATGTGTGGGTGTTTTTCTCTTTGTTTTTTACAGTACGGGAAGAATTCAGTGTAATAAACGTAACCGGTAATCGGATCAGGAACTTTTGGCTGCAGCTTTCACACAAGCAAGATTTAAAAATTAGATGTAGATCCTCTGTGGGTCGAGTCCTTCTGAAAGCATGCTGGATTTGGACTGAAACTAAGTTGTTAGTTATTTTCTAGCTTCCAGAGCAGCAGAGTGGGATTCACCGGCCTGTTTTGGGTCACCGTCGGTTCAGTCTTGAGGGGGTTCTGCTGGAAAACTGTGACGTCCTCTGGCCTGTTCTTGCCCAACTTTTCATCTAAACCAATAAATACTATTCAAGAC |
|  | Deduced amino acid sequence | MQSWTPLYRSLATRGHHVPSKLHGATALSVRTYADKAAIDADVTVVGSGPGGYVAAIKAAQLGFKTVCVEKNATLGGTCLNVGCIPSKALLNNSYFYHLAHGKDFESRGIEISGISLNLEKMMSQKSGAVKALTGGIAHLFKQNKVTHVSGLGRVSGKNQVTATGADGSEQVINTKNILIATGSEVTPFPGIHIDEETIVSSTGALSLKKVPEELIVIGAGVIGVELGSVWQRLGSKVTAVEFLGHVGGMGIDMEISKNFQRILQKQGLKFKLGTKVMGATKRPDGKIDVPVEAAAGGKNETLTCDVLLVCIGRRPYTQNLGLESVGIELDKRGRIPVNNRFQTQVPSIYAIGDVVAGPMLAHKAEDEGIICVEGMAGGAVHIDYNCVPSVIYTHPEVAWVGKTEEQLKEEGVPYKVGKFPFAANSRAKTNADTDGLVKILSHKETDRMLGSHILGSGAGEMINEAALAMEYGASCEDVARVCHAHPTVSEAFREANLAASFGKAINF |
| RFC3 | Nucleotide sequence | >TRINITY_DN5546_c0_g1_i2 GTGGTAATAAAGTCGATATTTAATCAAATCTATGAAAAAGAGAAAAAACAAACTTGAAAACATGTAAATCCTTTTTTGCCACTGGTTCAAGCAAAACTCCTGTTTGATATTTTGCAGCAGATCAAAGTTTGTAGCATCACTAACACCCGACCATGTACAGATGTTTATGCGACTGCAGGGAGACAGCTGGCAGTATTCTGGGGATGCAGCAGCAGCAGATTTCCTTGCAGCACTGGTCTCGTGGCCGTCTCGTGGCCTTTGAGCTCCTGCCAGAAATCAGAACATCATGGCATCCAGACCGTCCTCCATGAACTTCTTGTACATGGCCATGAACTTGGCCGTGAAGGCCTCCAGGTGGTAGATGGCCTTGTTGCCGAGCTGCAGTCTGTGTTCGTAGTAGGCCGCCATGTGGGCCACCTCCGTCTTCAGCTGGCCGTCGCAGTTACTGAGCAGCTCCTTCACCAGACCCTTCATGATGATGTCGGGGGGGATGCAGTGCGTCAGCAGCTCGTACAGCCGGGCCCGGACCTCCAACAGCCTCTGAGGACTCTGCTGGCTGACGATGGCGTTGGCCGTCTCTCTCAGGTAAACCTCCCAGTCCGTCTCTGGGACTTCCTGATCCACTGAGAACGGATACTGCTGAACCCTGCAGGCCTCGCACATCAGCAGAGCTTTGCGGAGGTTGCGGCCGGACTTGTCACTGATGTGTTTGGCCAGCTCCGGCGGCAGGAGCAGCCCCTCCTTCTTACAGACCGACGTCAGAACACTGCAGACCTCCTCGGTGCTGGGCAGAGGAACTCTGATGGCCAGGCAACGGCTTCTGATTGGCTCGATGACTTTGGAGGTGGAGGTGGAGCAGAGGATGAGCCGGCAGGTGGACATGTACTTTTCCATGGTCCGACGCAAGGCGTGCTGAGCGTCTTTGGTGAGTCGGTCCACCTCTGTTAGCAGAACCACTTTGAAGTCTCGCTGGCTGTTGGACTGGATCTGCTGGGACTGGGCCATGGTTTTGATCAGCTCCTGAATCACCACACGGTCCTGGTTCCCTGCATCACTTGCGTTGACTTCCAGGTGATAATTGCTGCTTATGGTGTTGATCTCAATCTTCTTCTTTGACGGGGCCACCACGGTCTGGTGCTCGATGCGCAGCTTCTCCACGCCGGCTCCGTACAGCTCCCTCAGCAGACACGTGATGCGGGTCTTCTTCCCGGCGCCGGCCGGCCCGTACACCAGCAGGTGGGGGAAGTCCCCGCACTGGACCAGGCTCTTCAGCTGGGCCGCCTGCTGCTTGTGGAAGTCCAGCTTTCCCAGGGAGCTGGGCCGGTACTTGTCCAGCCACAAACTCATCTTGCCGAAGGTTGGATGGTCCTGAGGGGTCGTGTGTCAGCAGGAGTTCGTCTGTCTGCAGGAGTCTTCTGTCTGCACCGCTGCTAGCCAATCTCCCGCGAAATGTTCATCCGCCCGCGCCGCGCATGCGCAGCGTCGGCAGCGCTACGGAAACGCGGGCGGGTCTTCTTTTC |
|  | Deduced amino acid sequence | MSLWLDKYRPSSLGKLDFHKQQAAQLKSLVQCGDFPHLLVYGPAGAGKKTRITCLLRELYGAGVEKLRIEHQTVVAPSKKKIEINTISSNYHLEVNASDAGNQDRVVIQELIKTMAQSQQIQSNSQRDFKVVLLTEVDRLTKDAQHALRRTMEKYMSTCRLILCSTSTSKVIEPIRSRCLAIRVPLPSTEEVCSVLTSVCKKEGLLLPPELAKHISDKSGRNLRKALLMCEACRVQQYPFSVDQEVPETDWEVYLRETANAIVSQQSPQRLLEVRARLYELLTHCIPPDIIMKGLVKELLSNCDGQLKTEVAHMAAYYEHRLQLGNKAIYHLEAFTAKFMAMYKKFMEDGLDAMMF |

| **Supplementary Table S2.** Oligonucleotide primer sequences and DNA preparation conditions | | | | | |
| --- | --- | --- | --- | --- | --- |
|  | Nucleotide sequence | Product size (nt) | Annealing temperature | Size selection condition* | Application |
| Cs-ACAT1-1st-PCR_f | GCAAAGCAGAGAGAAGCACA | 201 | 64°C | x0.8 and x1.2 | Generation of standard DNA |
| Cs-ACAT1-1st-PCR_r | TGGTACACAGCATTCAAGTCAG |  |  |  |  |
| Cs-DLD-1st-PCR_f | CGTTCAGAGAAGCCAACCTG | 208 | 64°C |  |  |
| Cs-DLD-1st-PCR_r | ACCACCTAAGCTGTTTCATCTT |  |  |  |  |
| Cs-RFC3-1st-PCR_f | GCCAGCTGAAGACGGAGG | 216 | 66°C |  |  |
| Cs-RFC3-1st-PCR_r | ATTTCCTTGCAGCACTGGTC |  |  |  |  |
| Cs-ACAT1-qPCR_f | AGCGAATGAATGTTGCCACTT | 69 | - | - | Quantitative PCR |
| Cs-ACAT1-qPCR_r | TTGAGGAGTTGCCTGAAAACAG |  | - | - |  |
| Cs-DLD-qPCR_f | ACGGGAGGCTGCGTTTCT | 60 | - | - |  |
| Cs-DLD-qPCR_r | TGGTGCAGGTTGGATGTACAG |  | - | - |  |
| Cs-RFC3-qPCR_f | TGGCGGCCTACTACGAACAC | 65 | - | - |  |
| Cs-RFC3-qPCR_r | GAAGGCCTCCAGGTGGTAGAT |  | - | - |  |

These primers were designed inside the putative last exon of the transcripts included in Supplementary Table S2.

*Concentrations for left-side and right-side size selection with AMPure XP beads are included.

**Supplementary Table S3**. RNA-seq library preparation and sequencing.

| Accession ID | Tissue | RIN | Number of PCR cycles | Number of raw reads obtained (million read pairs) |
| --- | --- | --- | --- | --- |
| DRR507760 | adult eyeball | 9.4 | 11 | 114.4 |
| DRR507761 | adult heart | 8.9 | 11 | 121.8 |
| DRR507762 | adult gill | 8.4 | 11 | 119.5 |
| DRR507763 | larvae whole | 9.8 | 11 | 108.5 |
| DRR507764 | adult muscle | 9.1 | 11 | 101.0 |
| DRR507765 | adult liver | 7.5 | 12 | 109.4 |
| DRR507766 | adult gut | N/A | 12 | 121.0 |

**Supplementary Table S4**. Aquaporin (AQP) gene entries used of molecular phylogeny inference.

| # in tree | Orthogroup^*^ | species | Accession ID |
| --- | --- | --- | --- |
| 1 | AQP8 | *Homo sapiens* | NP_001160.2 |
| 2 | AQP8 | *Danio rerio* | ACV60540.2 |
| 3 | AQP8 | *Cololabis saira* | This study |
| 4 | AQP8 | *Danio rerio* | NP_001073651.1 |
| 5 | AQP8 | *Danio rerio* | NP_001004661.1 |
| 6 | AQP8 | *Strongylocentrotus purpuratus* | XP_030854916.1 |
| 7 | AQP8 | *Patiria miniata* | XP_038075258.1 |
| 8 | AQP8 | *Patiria miniata* | XP_038069095.1 |
| 9 | AQP8 | *Strongylocentrotus purpuratus* | XP_030840396.1 |
| 10 | AQP11 | *Danio rerio* | AAH95775.1 |
| 11 | AQP11 | *Cololabis saira* | This study |
| 12 | AQP11 | *Cololabis saira* | This study |
| 13 | AQP11 | *Homo sapiens* | NP_766627.1 |
| 14 | AQP12 | *Danio rerio* | AAI21753.1_ |
| 15 | AQP12 | *Cololabis saira* | This study |
| 16 | AQP12 | *Homo sapiens* | NP_001095937.1 |
| 17 | AQP12 | *Homo sapiens* | NP_945349.1 |
| 18 | AQP11 | *Patiria miniata* | XP_038044509.1 |
| 19 | AQP11 | *Strongylocentrotus purpuratus* | XP_003725070.1 |
| 20 | AQP14 | *Cololabis saira* | This study |
| 21 | AQP14 | *Danio rerio* | XP_005174182.1 |
| 22 | cAQP | *Patiria miniata* | XP_038051349.1 |
| 23 | cAQP | *Strongylocentrotus purpuratus* | XP_001190612.2 |
| 24 | cAQP | *Patiria miniata* | XP_038078625.1 |
| 25 | cAQP | *Strongylocentrotus purpuratus* | XP_001185961.1 |
| 26 | AQP4 | *Danio rerio* | XP_009292895.1 |
| 27 | AQP4 | *Homo sapiens* | XP_011524244.1 |
| 28 | AQP4 | *Cololabis saira* | This study |
| 29 | AQP0 | *Cololabis saira* | This study |
| 30 | AQP0 | *Danio rerio* | NP_001018356.1 |
| 31 | AQP0 | *Danio rerio* | NP_001003534.1 |
| 32 | AQP0 | *Cololabis saira* | This study |
| 33 | AQP0 | *Homo sapiens* | NP_036196.1 |
| 34 | AQP5 | *Homo sapiens* | NP_001642.1 |
| 35 | AQP6 | *Homo sapiens* | NP_001643.2 |
| 36 | AQP2 | *Homo sapiens* | NP_000477.1 |
| 37 | AQP1 | *Homo sapiens* | NP_932766.1 |
| 38 | AQP1 | *Danio rerio* | XP_021336241.1 |
| 39 | AQP1 | *Danio rerio* | NP_996942.1 |
| 40 | AQP1 | *Cololabis saira* | This study |
| 41 | AQP15 | *Danio rerio* | XP_021327889.1 |
| 42 | glpAQP | *Patiria miniata* | XP_038071748.1 |
| 43 | glpAQP | *Patiria miniata* | XP_038073806.1 |
| 44 | glpAQP | *Patiria miniata* | XP_038073167.1 |
| 45 | glpAQP | *Strongylocentrotus purpuratus* | XP_792142.4 |
| 46 | glpAQP | *Strongylocentrotus purpuratus* | XP_030833261.1 |
| 47 | glpAQP | *Strongylocentrotus purpuratus* | XP_789770.3 |
| 48 | glpAQP | *Patiria miniata* | XP_038073505.1 |
| 49 | glpAQP | *Patiria miniata* | XP_038071359.1 |
| 50 | glpAQP | *Strongylocentrotus purpuratus* | XP_030832856.1 |
| 51 | AQP7 | *Cololabis saira* | This study |
| 52 | AQP7 | *Danio rerio* | NP_956204.2 |
| 53 | AQP7 | *Homo sapiens* | NP_001161.1 |
| 54 | AQP10 | *Danio rerio* | ACB10577.1 |
| 55 | AQP10 | *Cololabis saira* | This study |
| 56 | AQP10 | *Homo sapiens* | NP_536354.2 |
| 57 | AQP10 | *Danio rerio* | NP_001002349.1 |
| 58 | AQP10 | *Cololabis saira* | This study |
| 59 | AQP9 | *Homo sapiens* | NP_066190.2 |
| 60 | AQP9 | *Danio rerio* | NP_001028268.1 |
| 61 | AQP9 | *Cololabis saira* | This study |
| 62 | AQP9 | *Danio rerio* | NP_001171215.1 |
| 63 | AQP9 | *Cololabis saira* | This study |
| 64 | AQP3 | *Homo sapiens* | NP_004916.1 |
| 65 | AQP3 | *Danio rerio* | NP_998633.1 |
| 66 | AQP3 | *Cololabis saira* | This study |
| 67 | AQP3 | *Danio rerio* | NP_001159593.1 |

* Note that the gene product name in NCBI are often different from the gene name in this column which was inferred with molecular phylogeny inference.

**References**

1. Vurture, G. W., Sedlazeck, F. J., Nattestad, M., et al. 2017, GenomeScope: fast reference-free genome profiling from short reads. *Bioinformatics*, **33**, 2202–4.

2. Hu, B., Jin, J., Guo, A.-Y., Zhang, H., Luo, J., and Gao, G. 2015, GSDS 2.0: an upgraded gene feature visualization server. *Bioinformatics*, **31**, 1296–7.
